# A Web-Based Tool to Assess Social Inclusion and Support Care Planning in Mental Health Supported Accommodation: Development and Preliminary Test Study

**DOI:** 10.2196/45987

**Published:** 2024-03-13

**Authors:** Sharon Eager, Helen Killaspy, Joanna C, Gillian Mezey, Peter McPherson, Megan Downey, Georgina Thompson, Brynmor Lloyd-Evans

**Affiliations:** 1 Division of Psychiatry University College London London United Kingdom; 2 Camden and Islington NHS Foundation Trust London United Kingdom; 3 Population Health Research Institute St George's, University of London London United Kingdom

**Keywords:** social inclusion, supported accommodation, mental health, digital health, care planning

## Abstract

**Background:**

Individuals with severe mental illness living in supported accommodation are often socially excluded. Social inclusion is an important aspect of recovery-based practice and quality of life. The Social Inclusion Questionnaire User Experience (SInQUE) is a measure of social inclusion that has been validated for use with people with mental health problems. Previous research has suggested that the SInQUE could also help support care planning focused on enabling social inclusion in routine mental health practice.

**Objective:**

This study aims to develop a web-based version of the SInQUE for use in mental health supported accommodation services, examine its acceptability and perceived usefulness as a tool to support care planning with service users, determine the extent of uptake of the tool in supported accommodation settings, and develop a program theory and logic model for the online SInQUE.

**Methods:**

This study involved a laboratory-testing stage to assess the acceptability of the SInQUE tool through “think-aloud” testing with 6 supported accommodation staff members and a field-testing stage to assess the acceptability, utility, and use of the SInQUE tool over a 5-month period. An implementation strategy was used in 1 London borough to encourage the use of the SInQUE. Qualitative interviews with 12 service users and 12 staff members who used the tool were conducted and analyzed using thematic analysis. The use of the SInQUE was compared with that in 2 other local authority areas, 1 urban and 1 rural, where the tool was made available for use but no implementation strategy was used.

**Results:**

Overall, 17 staff members used the SInQUE with 28 different service users during the implementation period (approximately 10% of all service users living in supported accommodation in the study area). The staff and service users interviewed felt that the SInQUE was collaborative, comprehensive, user-friendly, and relevant. Although some staff were concerned that particular questions might be too personal, service users did not echo this view. Participants generally felt that the SInQUE could help identify individuals’ priorities regarding different aspects of social inclusion by prompting in-depth conversations and tailoring specific support to address service users’ inclusion goals. Some interviewees also suggested that the tool could highlight areas of unmet or unmeetable needs across the borough that could feed into service planning. The SInQUE was not used in the comparison areas that had no implementation strategy.

**Conclusions:**

The online SInQUE is an acceptable and potentially useful tool that can be recommended to assess and support care planning to enable social inclusion of people living in mental health supported accommodation services. Despite this, uptake rates were modest during the study period. A concerted implementation strategy is key to embedding its use in usual care, including proactive endorsement by senior leaders and service managers.

## Introduction

### Background

Social inclusion refers to an individual’s ability to participate in important societal activities and their sense of community belonging [1,2]. Someone may feel socially excluded if they do not have opportunities for societal involvement and integration, often because of external factors that are beyond their control [3]. Social exclusion is a multifaceted continuum [2], typically signified by poverty, unemployment, inequality, and poor health [4].

People with serious mental illness are thought to be among the most socially excluded groups in society [5]. Individuals with this type of mental health problems often have smaller and less satisfying social networks [6], lower household income [7], and lower levels of employment [8,9] and experience more criminal and violent victimization [10,11] than those in the general population. Social exclusion can be conceptualized as both a cause and a consequence of mental illness [12]. Furthermore, greater social inclusion is associated with better quality of life and lower levels of loneliness among those with severe mental illness, suggesting that social exclusion is an important area for mental health practitioners to try to address [13,14].

Mental health supported accommodation services provide care and support to individuals with particularly severe and complex mental health problems as a way of supporting recovery in the community [15]. It is estimated that there are approximately 100,000 people living in mental health supported accommodation in England. Services are typically staffed by support workers, with additional specialist clinical input provided by National Health Service (NHS) community mental health teams [16]. In England, three main types of supported accommodation are provided: (1) residential care homes for those with the highest needs that comprise 24-hour–staffed communal facilities where placements are not time limited, with meals, supervision of medication, cleaning, and activities provided to service users; (2) supported housing services that provide shared or individual, self-contained, and time-limited tenancies with staff based on-site up to 24 hours a day to assist service users in gaining skills to move on to less supported accommodation; and (3) floating outreach services that provide visiting support for a few hours per week to people living in permanent, self-contained, and individual tenancies with the aim of reducing support over time to zero [16].

Service users living in supported accommodation are often socially isolated, with low levels of employment and little involvement in civil and political processes [17]. Many report feeling lonely and isolated and experiencing a high level of stigma that causes them to become more socially isolated [18]. There is evidence that users of mental health supported accommodation services report a variety of unmet needs, such as accessing employment opportunities and forming intimate relationships [19,20]. However, relatively little research has been conducted to determine the precise needs of service users living in supported accommodation [21], and a greater focus on this group is needed to identify and implement interventions that are likely to be the most useful for them [22].

Supporting service users to work toward desired goals and community engagement is highly congruent with recovery-based practice in mental health. Recovery-based practice recognizes and builds on service users’ strengths and promotes empowerment through collaboration between them and staff to identify and work toward specific goals [23]. Many of the identified goals are markers of social inclusion, such as employment, social network development, and participation in community activities [24]. There is qualitative evidence from a large national research program suggesting that staff working in mental health supported accommodation services operate with a considerable degree of recovery orientation [24,25], and the more recovery-orientated these services are, the more likely people are to move on successfully to more independent settings [26].

People living in mental health supported accommodation have expressed a strong preference for individually tailored services that offer choice and promote autonomy, consistent with a recovery-based approach [18]. Patient-reported outcome measures have been recommended to inform this individualized approach by directly capturing service users’ perspectives on constructs such as goal attainment, quality of life, and social inclusion [27]. Such measures enable service users to make informed decisions about their own support and care planning, in line with World Health Organization recommendations for recovery-based practice in community care provision [28]. Resources delivered across web-based platforms, particularly those that offer guided support, have been established as accessible, acceptable, and effective for use by participants with severe mental illness [29,30]. A tailor-made web-based assessment tool, the Quality Indicator for Rehabilitative Care for supported accommodation, has also been successfully used by managers of supported accommodation services, suggesting that these settings have the required resources and expertise to implement online measures [31].

The Social Inclusion Questionnaire User Experience (SInQUE) was developed as a measure of social inclusion for individuals with severe mental illness [32]. The measure has been validated across a range of mental health populations, has established reliability, is considered acceptable to service users, and has been proposed as being potentially cross-culturally suitable [32-34]. To date, the SInQUE has been used solely in offline research contexts. However, stakeholder feedback from a previous study testing the SInQUE indicated that the measure may be useful in clinical practice to assess social inclusion, facilitate important conversations with service users, and guide care and support planning [34]. Furthermore, a consistent research recommendation from the developers of the SInQUE tool has been to investigate whether the measure has utility as a care-planning tool to promote social inclusion in routine mental health practice [13,34].

This study aimed to develop a web-based version of the SInQUE for use in mental health supported accommodation services. We sought to examine the acceptability and perceived usefulness of this tool among supported accommodation staff and service users as a means to assess their needs for greater social inclusion and promote care planning.

### Aims

The study aims were as follows:

1. To develop and refine a web-based version of the SInQUE social inclusion assessment tool tailored for use in mental health supported accommodation settings.
2. To investigate the acceptability and perceived utility of the online SInQUE tool among supported accommodation staff and service users.
3. To determine the extent of uptake of the tool in supported accommodation settings with and without a locally developed implementation strategy to support its use.
4. Informed by the study findings, to develop a program theory and logic model for the online SInQUE specifying its anticipated outcomes, the mechanisms through which they may be achieved, and contextual factors affecting the use and experience of the SInQUE.

## Methods

### Study Design

This study comprised two stages conducted in 1 inner London borough:

1. A laboratory-testing stage to assess initial acceptability of the tool and develop it through “think-aloud” testing and semistructured interviews with supported accommodation staff.
2. A field-testing stage to assess wider acceptability, feasibility, and use of the tool. Semistructured interviews were conducted with staff and service users who had used the online SInQUE during this stage.

The 5-month field-testing stage was supported by a local implementation strategy developed in collaboration with local service leads to support the use of the online SInQUE by supported accommodation staff in the participating London borough. The online SInQUE was also made available to supported accommodation services by local service leads in 2 other areas without any accompanying implementation strategy.

### Description of the SInQUE Tool

The web-based version of the SInQUE [35] can be used to assess social inclusion and inform support and care planning for people with mental health problems. It is designed to be used by staff as part of routine care planning to be completed collaboratively with service users. It can be used on a computer, tablet, or mobile device. Staff are required to register for an account on the SInQUE site using their work email address and details of their organization and can then use the tool for free. No personal data identifying service users are logged or stored on the SInQUE platform. The online SInQUE generates a unique reference number for each new service user, which is retained by the staff member completing the assessment for future reference and to link any repeat assessments.

The online tool developed for laboratory testing in our study included the 46-item version of the validated SInQUE social inclusion questionnaire, which was refined following stakeholder feedback at the end of the previous measure development study [34]. For this study, we removed 1 question from the SInQUE that asked whether the respondent was living alone as this was considered redundant for people living in residential care and supported housing. The SInQUE’s psychometric properties have been established among people with a range of mental health problems receiving input from community mental health services in previous studies [13,32,34]. Although the removal of a question from the original SInQUE questionnaire compromised its established psychometric properties, this minor adaptation is unlikely to have disrupted them substantially. We wanted to minimize changes to maintain the online SInQUE’s similarity to the validated measure and did not aim to make further significant refinements to the content of the tool. Instead, we wanted to gain feedback on its acceptability and feasibility for use in its digital format among staff and users of mental health supported accommodation services to assist in care and support planning.

The online SInQUE questionnaire yields a total score of 0 to 75, with a higher score indicating greater social inclusion. The questions and subscale scores are grouped into 9 different areas of social inclusion: leisure, social relationships, religious and cultural activities, education and employment, transport, health, crime victimization, home life and housing, and civic duties. These areas cover the 5 social inclusion domains of the validated SInQUE (social integration, productivity, consumption, access to services, and political engagement), but the aforementioned 9 areas were considered more immediately understandable for use in practice.

Using the service user’s responses, the online SInQUE generates a list of areas in which the person has said that they would like to be more socially included. It then offers a prompt for the service user and staff member to collaboratively select up to 3 priority areas that they would like to integrate into the person’s support plan. Once the assessment is completed, a summary report is generated. If the assessment is repeated with the same service user in the future, this report will also display changes in their social inclusion over time. The tool can generate management-level summary reports for each organization that is registered with it and commissioner-level summary reports of services using the tool across an entire area (such as a London borough). Multimedia Appendix 1 provides a full description of the SInQUE using the TIDieR (Template for Intervention Description and Replication) checklist [36].

### Setting

This study took place in mental health supported accommodation services across 1 inner London borough. There are 21 such services in the borough run by 6 different voluntary sector organizations. They offer varying degrees of support to >270 service users who are also supported by local NHS secondary mental health services. In the borough, there are approximately 24 service users living in residential care, 159 living in supported housing, and 89 who receive floating outreach support. Supported housing services offer 24-hour support to 119 people and “9 to 5” support to 40 individuals.

### Laboratory-Testing Stage: Recruitment, Data Collection, and Analysis

In total, 6 supported accommodation staff members were recruited to provide initial impressions of the online SInQUE tool. We discussed the study with service managers working in 3 different services and asked them to nominate 2 staff members each from their service who were interested in taking part. Participants were purposively sampled to include staff working in floating outreach support, 24-hour supported housing, and residential care. We asked the managers of each of the 3 services to ask for volunteers from their staff teams. Interviews were arranged with the first 2 staff members identified by the manager of each service.

Data were collected between January 2022 and February 2022. All 6 think-aloud interviews with staff members were conducted and recorded using Microsoft Teams (Microsoft Corp). The researcher first discussed the information sheet with each participant and gave them the opportunity to ask questions. Following this, participants’ consent was verbally collected and audio recorded separately from the main part of the interview. Participants were then asked to fill out a short online form providing their demographic information before beginning the interview.

We conducted “think-aloud” testing of the online tool with staff using a semistructured topic guide developed by the study team (Multimedia Appendix 2). Following a process used previously in developing web-based tools [37], participants were asked to complete set tasks using the online SInQUE tool while providing a continuous commentary on their thoughts. They were asked to open the SInQUE website, register for an account, and complete an assessment as they would with a service user. At all stages, they were prompted to share their thoughts as they navigated the website and offer their initial impressions on how easy it was to understand and use and its potential suitability for their work. Once participants had completed the questionnaire, the researcher asked broader questions about their experience using the tool and any ways in which it could be improved. Throughout these interviews, participants were asked to focus on their experience using the SInQUE tool rather than offering specific feedback on individual SInQUE items. This was because we did not intend to make substantial modifications to the SInQUE questions to maintain their scope and similarity to those of the validated SInQUE measure.

Identified problems and suggestions for improvements to the online tool were collated by the researcher following the interviews. They were then reviewed by the study team, decisions about refinements to the online SInQUE were agreed upon, and the tool was revised accordingly.

### Field-Testing Stage: Recruitment, Data Collection, and Analysis

#### Local Implementation Strategy

The revised version of the tool was made available for use in mental health supported accommodation services across the participating inner London borough. We iteratively developed and implemented a strategy to encourage and support its use by supported accommodation staff in the borough over a 5-month period beginning on May 11, 2022. This implementation strategy was informed by consultation with supported accommodation service managers and clinicians working in the Islington community mental health rehabilitation team and by individual interviews conducted with supported accommodation service users and staff.

#### Interviews With Field-Testing Participants

##### Participants and Recruitment

Individual interviews with service users (n=12) and staff (n=12) who tried out the SInQUE tool were conducted from late May 2022 to September 2022. This number was chosen to explore the views of staff and service users from a variety of supported accommodation types and service providers. Following our implementation strategy, we asked staff members to alert the study researcher once they had tried the tool in practice. Any staff member or service user in supported accommodation who tried the SInQUE tool was eligible to participate in an individual interview.

Once a staff member informed the study researcher that they had tried the tool, we asked them whether they would like to participate in an individual interview about their experience. We also invited staff to pass on information about the study to the service users with whom they had used the tool and ask them whether they would like to participate in an interview about their experience. If the service user was interested in taking part, the researcher communicated with them either directly or through the staff member they had completed the SInQUE assessment with depending on their preference. Toward the end of the recruitment stage, we recruited the final few staff members and service users purposively to ensure that participants were from a range of supported accommodation types and provider organizations.

One service user interview and 1 staff member interview were conducted online via Microsoft Teams; all other interviews were carried out in person according to participants’ preferences. In-person interviews were conducted by the study researcher at the staffed supported accommodation sites, aside from 1 interview with a service user receiving floating outreach support, which was conducted at their home.

##### Measures and Procedures

The researcher first discussed the information sheet with the participants and gave them the opportunity to ask questions about the study. For in-person interviews, informed consent was collected via a paper consent form; for online interviews, verbal consent was audio recorded. Participants were then asked to answer brief demographic questions about themselves and their associated services. Following this, the researcher asked each participant questions about their experience using the SInQUE; whether there were any ways in which it could be improved; the appropriateness of the online tool for use in their work; and what impacts, if any, they thought it might have on care provision and service users’ experience. The interview topic guides (one for staff participants and one for service user participants) were developed by the study team as semistructured interviews—they are provided in Multimedia Appendix 3. In-person interviews were recorded using a digital voice recorder; online interviews were recorded on Microsoft Teams. Interview audio recordings were transcribed by a professional transcription company with which University College London (UCL) had a data-sharing and privacy agreement. Interview transcripts were then checked by the study researcher for accuracy. Any potentially identifiable text was anonymized. The resulting cleaned transcripts were then securely stored on the UCL university system.

##### Analysis

The analysis of the interviews comprised 2 stages. First, the study researcher noted any problems experienced by participants and recorded improvements to the online SInQUE they suggested. These issues and the suggested changes were reviewed by the study team, as in the previous laboratory-testing phase. Minor modifications to the online SInQUE were agreed upon, and we made adjustments to the tool in line with this.

Second, transcripts were uploaded to NVivo (version 12; QSR International) for qualitative analysis. As we aimed to develop a program theory for the online SInQUE intervention, we initially coded data into a deductively derived framework that used an intervention-context-actor-mechanism-outcome (ICAMO) configuration, with each component of this ICAMO framework representing a primary theme [38]. Within each of these 5 primary themes, we inductively derived subthemes from the data using thematic analysis. The initial coding was conducted by the lead author (SE) and was then reviewed and adjusted collaboratively by the study team. This included gaining lived-experience perspectives from a researcher with experience of mental health service use (JC) and clinical insights from a senior clinical academic working in the participating borough as a consultant rehabilitation psychiatrist supporting service users who live in supported accommodation (HK). The team brought in further perspectives from those with backgrounds in social work (BLE), clinical psychology (PM), and forensic psychiatry (GM) and from the community rehabilitation team in the borough (MD).

#### Data Use Monitoring

Data on the uptake and use of the online SInQUE tool were collected from the online SInQUE informatics for the 5-month field-testing period from May 11, 2022, to October 11, 2022.

At the start of this period, the study team also contacted local mental health service rehabilitation and housing leads in 2 other areas: another inner London borough and a rural county in the west of England. These service leads contacted local supported accommodation managers and invited them to use the online SInQUE in their service if they wished. The tool was made available to 7 supported accommodation services in the London borough and 10 in the rural county. No further encouragement to use the tool or implementation support was provided. This allowed us to monitor uptake and use of the tool in 2 areas without an associated implementation plan, thus making inferences about the necessity and impact of the strategy we developed.

#### Logic Model Development

The study team developed a preliminary logic model for the online SInQUE in planning this study. We used the findings of the aforementioned research activities to review and refine this logic model and develop an updated theory about the potential outcomes for service users and organizations from using the online SInQUE; mechanisms through which these outcomes are achieved; and factors influencing the uptake, experience, and impact of the online tool. Factors were related to (1) the intervention itself, (2) the characteristics and attitudes of staff and service users using the online SInQUE, and (3) the broader organizational and societal context. This was summarized in a logic model in the form of an “ICAMO map” [38], which was developed and refined iteratively through discussion with the study team.

### Ethical Considerations

The initial laboratory-testing phase of this study (Supporting social inclusion for people with serious mental illness living in supported housing [SUSHI] phase 1) was approved by the UCL Research Ethics Committee (REC) on June 18, 2021 (REC reference 6711/002). The subsequent field-testing phase (SUSHI phase 2) was approved by the London – Camden and Kings Cross NHS REC on November 4, 2021 (REC reference 21/LO/0657). Written or audio-recorded informed consent was obtained from all participants before they took part, and they were clearly informed that they could opt out of the study at any time. All the study data were carefully deidentified. Service user participants were offered a £20 (US $25.14) shopping voucher to thank them for their time.

## Results

### Participants

We recruited 6 supported accommodation staff members for the “think-aloud” interviews during the laboratory-testing stage. We recruited a further 12 staff members and 12 supported accommodation service users for the individual interviews as part of the field-testing stage. Participant characteristics for both stages are summarized in Table 1.

**Table 1:** Laboratory testing and field testing of the online Social Inclusion Questionnaire User Experience—characteristics of participants.

Participant characteristics		Laboratory testing	Field testing	
		Staff (n=6), n (%)	Staff (n=12), n (%)	Service users (n=12), n (%)
**Gender**				
	Male	2 (33)	5 (42)	11 (92)
	Female	4 (67)	6 (50)	0 (0)
	Nonbinary	0 (0)	1 (8)	1 (8)
**Age group (y)**				
	18-30	0 (0)	8 (67)	4 (33)
	31-50	2 (33)	2 (17)	3 (25)
	≥51	4 (67)	1 (8)	4 (33)
	Prefer not to say	0 (0)	1 (8)	1 (8)
**Ethnicity**				
	Asian/Asian British	1 (17)	0 (0)	1 (8)
	Black/Black British	3 (50)	2 (17)	4 (33)
	White/White British	2 (33)	8 (67)	1 (8)
	Mixed/multiple ethnic groups	0 (0)	1 (8)	6 (50)
	Other ethnic background	0 (0)	1 (8)	0 (0)
**Sexual orientation**				
	Heterosexual	N/A^a^	N/A	6 (50)
	Gay/lesbian	N/A	N/A	1 (8)
	Bisexual	N/A	N/A	2 (17)
	Prefer not to say	N/A	N/A	3 (25)
**Type of supported accommodation lived or worked in**				
	Floating outreach support	2 (33)	2 (17)	2 (17)
	9-to-5 supported housing	2 (33)	1 (8)	2 (17)
	24-h supported housing	2 (33)	7 (58)	8 (67)
	Residential care	0 (0)	2 (17)	0 (0)
**Length of time worked or lived in supported accommodation (y)**				
	<2	1 (17)	7 (58)	5 (42)
	2-5	3 (50)	3 (25)	5 (42)
	6-10	1 (17)	2 (17)	0 (0)
	≥10	1 (17)	0 (0)	1 (8)
	Prefer not to say	0 (0)	0 (0)	1 (8)

^a^N/A: not applicable; staff were not asked about their sexual orientation.

### Changes Made to the SInQUE

Following phase 1 laboratory testing and phase 2 field-testing, suggestions that participants made for how the tool could be improved were collated and reviewed by the team. Accordingly, adjustments were made to the online SInQUE after each stage, an overview of which can be found in Table 2. This addressed aim 1 of this study.

**Table 2:** Changes made to the online Social Inclusion Questionnaire User Experience (SInQUE) following phase 1 and phase 2 testing. All changes were made following the initial laboratory-testing stage unless indicated otherwise.

Section of the SInQUE affected		Explanation of the problem	Resolution	Justification for the change
**Registration and use changes**				
	The online SInQUE home page	Some staff members and service users suggested developing additional materials to help explain the SInQUE^a^.	Developed a guidance manual for service managers and commissioners with information on using the SInQUE as well as an informational leaflet and poster aimed at service users about the SInQUE.	It is easier for managers, commissioners, staff, and service users to understand and use the SInQUE.
	The initial page where staff members are asked to register for the SInQUE	Staff were asked to enter the organization they worked for in a free-text box. Some found it confusing to know which organization name they should enter.	Changed response options to a fixed-response drop-down menu with all housing providers in the borough and an “other” free-text option.	Allows for compilation of service-level data and is easier for staff to navigate.
	The page where staff members enter details to set up a new SInQUE assessment	Some staff members thought that further information on the exact purpose of the questionnaire and how it should be administered would be useful in the introductory paragraph describing the assessment.	Additional guidance on how the questionnaire should be administered was added to the introduction paragraph of the SInQUE.	Important contextual information for the questionnaire was explicitly clarified.
	The page where staff members enter details to set up a new SInQUE assessment	Some staff members found the wording of the following question—“Please select the type of accommodation in which the service user is living from the list below”—to be ambiguous and confusing.	Changed the wording of the question to the following: “Please select the type of housing support the service user receives from the list below.”	The clarity of the question improved.
**Changes to the wording of SInQUE questions**				
	The section covering “leisure” questions	Some staff members felt that it was unclear what the “Other” option meant in the context of question 3f: “Over the past year have you been to...Other?”	Changed the wording of this subquestion to the following: “Other leisure activity?”	The clarity of the question improved.
	The section covering “leisure” questions	Some staff members and service users felt that question 7—“Do you spend time in pubs or cafés?”—was worded in a way that was potentially inappropriate for people who do not drink alcohol^a^.	Changed the wording of the question to the following: “Do you go out for a coffee/drink (e.g. to a café or pub, etc) at least once a week?”	The clarity and appropriateness of the question improved.
	The section covering “social” questions	Some staff members were unsure whether question 9—“How many people, outside those in your care team, could you confide in?”—related to a professional or personal care team.	Changed the wording of the question to the following: “How many people, outside the workers in your care team, could you confide in?”	The clarity of the question improved.
	The section covering “home life/housing” questions	Some staff members thought that question 36—“What kind of accommodation do you live in?”—was worded ambiguously.	Changed the wording of the question to the following: “What type of housing support do you receive?”	The clarity of the question improved.
	The section covering “home life/housing” questions	A statement alerting users that question 38 had been omitted from the online SInQUE, which read the following—“Not relevant for supported accommodation contexts -omitted*.*”*—*was confusing.	Changed statement to the following: “Question omitted, not included in the online SInQUE.”	The clarity of the question improved.
**Changes to the SInQUE summary outputs**				
	The SInQUE summary report	Some staff members found the spider graph to be confusing to interpret as the numbers summarizing scores in each section were not standardized and, therefore, it was difficult to tell which domains scored lower than others.	Simplified the spider graph to show percentage of total score in the graph instead of frequency.	It was easier to interpret the graph as sections with different totals became standardized.
	The SInQUE summary report for multiple assessments completed with the same service user	In the section summarizing scores across multiple time points, staff members thought that a visual depiction of this comparison would be useful.	Made comparative bar charts of multiple scores across time with the same service user available on the summary report.	It was easier to understand and relay the results.

^a^Changes were made following the field-testing stage.

Overall, the changes made to the online SInQUE were relatively few and minor. Following initial laboratory testing, additional information and guidance for users was added, and minor revisions to the wording of questionnaire items were made to improve clarity. Modifications to the visual representation of scores in the summary reports were also made to aid ease of interpretation. During the field-testing stage, very few suggested changes to improve the usability of the online SInQUE were made by staff or service user participants. Further changes made at this stage included a minor wording adjustment to one question to ensure its cross-cultural appropriateness. Changes were made exclusively to the web-based version of the SInQUE and did not affect the existing SInQUE measure. We also developed an additional guidance document for managers and commissioners and an informational leaflet and poster about the SInQUE.

A few participants suggested substantial modifications to the structure and wording of individual items in the SInQUE that were not implemented by the study team. These decisions were made to preserve the broad scope and logical flow of the tool. We also declined to action some suggestions that were outside the remit of the SInQUE tool, such as adding more or free-text response options to some questions. However, where these suggestions indicated important potential barriers to using the SInQUE, they were noted and integrated into the qualitative analysis and logic model development. A summary of all comments and suggestions that were proposed but not implemented after review by the team can be found in Multimedia Appendix 4.

### Interview Thematic Analysis

#### Overview

The interviews were analyzed using thematic analysis. Primary themes were deductively imposed according to each core element of the ICAMO model: intervention, context, actors, mechanisms, and outcomes [38]. Subthemes were inductively analyzed within each of these primary themes. The resultant thematic framework considering the perceived utility and acceptability of the online SInQUE and addressing aim 2 of the study is presented in Textbox 1. The themes are summarized in the following sections with a selection of illustrative quotes. Multimedia Appendix 5 provides further illustrative quotes for each subtheme.

**Summary of the thematic framework (intervention-context-actor-mechanism-outcome themes and inductive subthemes).**

**Intervention: combination of program elements or strategies designed to produce behavior changes or improve health status among individuals**

- The online Social Inclusion Questionnaire User Experience (SInQUE):
  Promotion of positive, collaborative discussion
  Comprehensive and novel questions
  Ability to repeat over time
  User-friendly design:
    Easy-to-navigate website
    Quick to complete
    Fixed-response questions
    Offers options to choose from
    Web-based format

**Context: salient conditions that are likely to enable or constrain the activation of program mechanisms**

- Relevance of the SInQUE to staff role
- Inconsistency in current assessments used across services
- Absence of comparably specific assessments
- Emergence from the pandemic

**Actors: the individuals, groups, and institutions who play a role in the implementation and outcomes of an intervention**

- Staff:
  Professional knowledge and skills
  Professional boundaries
- Staff (service user views about staff):
  Trusting relationship
  Proactivity in offering guidance and support
- Service users:
  Familiarity and comfort with the questions
  Individual language and cultural differences of service users
- Service users (staff views about service users):
  Engagement in the assessment
  Existing mental health needs

**Mechanisms: any underlying determinants or social behaviors generated in certain contexts**

- Using the online SInQUE can accomplish the following:
  Boost service user proactivity and confidence
  Identify service users’ priorities on social inclusion
  Prompt novel, personal conversations
  Monitor changes in social inclusion over time
  Identify gaps in support available within the organization and local community

**Outcomes: behavior changes that follow the immediate knowledge change (intermediate) and changes such as patients’ health status and impact on community and health system (long term)**

- Intermediate:
  Improve staff relationship with and understanding of the service user
  Help plan more relevant, targeted support for the service user
- Long term:
  Borough-level improvements and changes in services to support social inclusion
  Individual-level benefits for service users’ recovery and social inclusion

#### Intervention

In general, staff and service user participants felt that the tool was user-friendly and collaborative. Many noted the ability to repeat the assessment and the web-based format as being particularly useful and felt that the website was easy to navigate. The short length of the assessment was also discussed as an important advantage, with both staff and service users commenting that it felt quick to fill out. Participants noted that, despite the short assessment length, it still offered a range of interesting, positive, and sometimes unfamiliar questions that felt comprehensive and useful to discuss:

I think that it wasn’t too just baseline, it was a little bit more than that and I think that’s good. Because it gives the option of, “Okay, you don’t want something, how can we improve and what is it that you do want that could help you while you’re in our service?”

212; staff member

Interviewees felt that the user-friendliness was aided by accessible questions that were straightforward for service users to answer and that were cross-culturally appropriate for individuals from different ethnic backgrounds. Although some participants from both groups felt that the fixed-response options for the questions were limited, certain staff members thought that this made the questionnaire more accessible to service users who may otherwise struggle with engagement.

#### Context

Staff members largely felt that the tool was fitting and relevant to their role in helping support service users, and most did not already use assessments that were highly similar to the SInQUE. Certain staff members highlighted a lack of continuity of support workers within their service and noted that this often made it difficult to build rapport with service users. Some also commented on an inconsistency in assessments used across different services (in the local context, where 6 different provider organizations provided supported accommodation services across the borough). They noted that individual providers currently make their own recommendations on the tools that staff should use:

If it was a standard central assessment that we do in all supported housing, that’s similar, like this for example, it might be beneficial in the long run. But each company has their own policy around it.

205; staff member

One staff member noted that the tool felt particularly relevant following the COVID-19 pandemic as a means to promote engagement among service users after a period of likely sustained social isolation.

#### Actors

There were 2 key actors to consider in the application of the assessment: the staff members who asked the questions and the service users who responded to them.

One staff member felt that the assessment was not particularly relevant in the context of their work in a residential care service, where they had an established relationship with service users and already knew much of the queried information about them. However, this was an outlying view. Although most staff members thought that the online tool could be suitable and useful for their work, they emphasized the importance of using their professional knowledge and skills to pick when and for whom the assessment would be appropriate. They suggested that service users acutely struggling with their mental health may find it difficult to maintain concentration and engagement with the questionnaire and others may feel that the assessment is not relevant to them.

Staff members also raised the importance of maintaining professional boundaries with service users, and some expressed a concern that certain questions may feel invasive or uncomfortable for service users to answer:

I think there was one quite private like about if they’re in a relationship or something, and that was the only question that made me feel a bit like I’m asking something very personal about a relationship. Because they might not want to say that.

210; staff member

However, service user participants did not express any similar concerns about intrusive questions. They generally indicated that they felt comfortable with the assessment and that they were used to answering personal questions. Both staff and service users highlighted trust between those performing the assessment as a key factor in promoting engagement with such questions.

Both respondent groups highlighted the cultural diversity of service users within supported accommodation, and many noted that the tool felt appropriate for those from a variety of religious and ethnic backgrounds. Some service users commented on the importance of the staff members being proactive and taking the time to go through the assessment in detail with them, particularly individuals for whom English was a second language, as further explanation was required for some questions. Various staff members suggested that being provided with information about the assessment and its purpose specifically aimed at service users, for instance, a guidance leaflet, would be helpful for them to convey the essential information about the assessment.

#### Mechanisms

Both participant groups discussed how the tool may boost confidence and proactivity for a wide range of service users by highlighting specific, achievable ways in which an individual can improve their social inclusion. They also noted how the assessment encourages service users to open up and enables more profound conversations between them and staff members:

Yes I found it really interesting, so like because it’s not really topics I would actually talk about. So it gave me a bit of enthusiasm to talk about some of the questions.

409; service user

Both groups suggested that it might be particularly useful during key working sessions as a means to get to know an individual better and identify their support preferences soon after moving into supported accommodation. Participants also noted the value of repeating assessments over time, suggesting that this could be a potentially encouraging way to demonstrate service user progress and identify gaps in available support. The most frequently suggested time between assessments was 1 to 3 months, with up to 6 months mentioned as a potential maximum gap.

#### Outcomes

Interviewees discussed the short- and long-term outcomes that they felt the tool could offer. They discussed how the tool enabled targeted and relevant support that prioritized the service user’s interests. Both groups also mentioned the potential for the tool to improve the relationship and understanding between service users and staff members:

It asks questions where maybe like for your support worker to get a better understanding of you, like even though the immediate thing is highlight areas you can work on, it gives a general overview of how you are.

410; service user

Some staff members also discussed how prolonged use of the tool could highlight the additional borough-level support that may be needed to improve certain gaps in support and could also promote service user recovery toward the goal of more independent accommodation.

### Implementation Strategy

Our implementation strategy was developed to encourage the use of the SInQUE in the supported accommodation services in the borough and was updated through consultation with clinical staff working in the borough’s community rehabilitation team and supported accommodation service managers. Our strategy was further informed by feedback from staff and service user participants during both stages of the study.

Each part of the strategy was developed to target an identified potential barrier to staff using the online SInQUE with service users. Subsequently, we mapped each component of the strategy to the 3 broad domains of the Capability, Opportunity, and Motivation–Behavior framework of behavior change [39] to describe whether each element of the strategy was intended to increase the staff’s capability, opportunity, or motivation to use the online SInQUE. The complete implementation strategy and the Capability, Opportunity, and Motivation–Behavior domain that each component addressed are outlined in Table 3. Strategies were related to enlisting leadership support to encourage supported accommodation staff to use the SInQUE, providing technical guidance and assistance with using the online tool, and developing bespoke summary output reports to reinforce use and increase the organizational benefits of using the SInQUE.

**Table 3:** Summary of the implementation strategy to support the use of the online Social Inclusion Questionnaire User Experience (SInQUE).

Activity	Implementation goal being addressed	COM-B^a^ domain
Study research assistant (SE) and clinical research staff member (MD) visit all supported accommodation services to introduce the SInQUE tool to staff and offer guidance on its use.	Increase awareness of the SInQUE tool among supported accommodation staff and respond to any of their concerns or other problems.	Motivation Opportunity Capability
NHS^b^ community rehabilitation team manager and clinical researcher (MD) review supported accommodation caseloads to identify suitable service users for a SInQUE assessment and ask their key worker to complete an assessment with those service users.	Lack of accountability after initially asking all staff members to use the SInQUE and staff hesitancy over which service users would be suitable for an assessment	Motivation
NHS community rehabilitation team manager contacts supported accommodation managers to ask them to support and encourage the use of the SInQUE by identified key workers within a given time frame.	Lack of supported accommodation management prioritization for staff to use the SInQUE	Motivation Opportunity
Local authority commissioners contact all supported accommodation managers to encourage the use of the SInQUE within services in the borough.	Lack of supported accommodation management prioritization for staff to use the SInQUE	Motivation
Study lead (BLE) and research assistant (SE) attend local Housing Forum meetings to update on the study and encourage the use of the SInQUE among all managers and staff members present.	Increase visibility and awareness of the SInQUE among supported accommodation managers and respond to any of their concerns or other problems.	Motivation Opportunity
Study research assistant (SE) offers supported accommodation staff technological support with SInQUE registration and use.	Uncertainty about how to manage the technical process of using the SInQUE	Capability
Study team develops and circulates a leaflet about the SInQUE for supported accommodation staff to give to service users to help explain the purpose of an assessment.	Uncertainty among some supported accommodation staff members about how best to explain the purpose of the SInQUE and engage service users	Capability
Study team sends summary reports to service managers outlining use of the SInQUE and highlighting the areas of social inclusion that are most frequently prioritized and addressed in their service^c^.	Increased awareness among supported accommodation managers of the value offered by the SInQUE for service planning to encourage them to prompt staff to use it.	Motivation
Study team sends summary reports to local authority commissioners outlining which services have used the SInQUE the most and highlighting the areas of social inclusion that are most frequently prioritized and addressed across all services in the borough^c^.	Increased awareness of commissioners of the value offered by the SInQUE for service planning and commissioning to encourage them to prompt services to use it.	Motivation

^a^COM-B: Capability, Opportunity, and Motivation–Behavior.

^b^NHS: National Health Service.

^c^These actions were planned with service managers and commissioners but not carried out during the 5-month implementation period because of the small number of completed SInQUE assessments.

### Usage Data

In total, 27 staff members in the inner London borough registered for an account with the online SInQUE. Of the 27 staff members who registered, 17 (63%) from 6 different supported accommodation providers started or completed a SInQUE assessment with at least 1 service user. This resulted in 30 completed SInQUE assessments with 28 service users in the borough. This represented just >10% of the estimated total number of service users living in supported accommodation in the borough. Of the 28 service users, 4 (14%) were from residential care, 19 (68%) were from 24-hour supported housing, 3 (11%) were from 9-to-5 supported housing, 1 (4%) was from floating outreach services, and 1 (4%) was registered as “other” accommodation type. One staff member from 1 of the local authority areas where there was no specific implementation strategy registered for an account with the SInQUE; however, they did not start or complete a SInQUE assessment. This addressed aim 3 of the study.

### Intervention Logic Model

**Figure 1:**
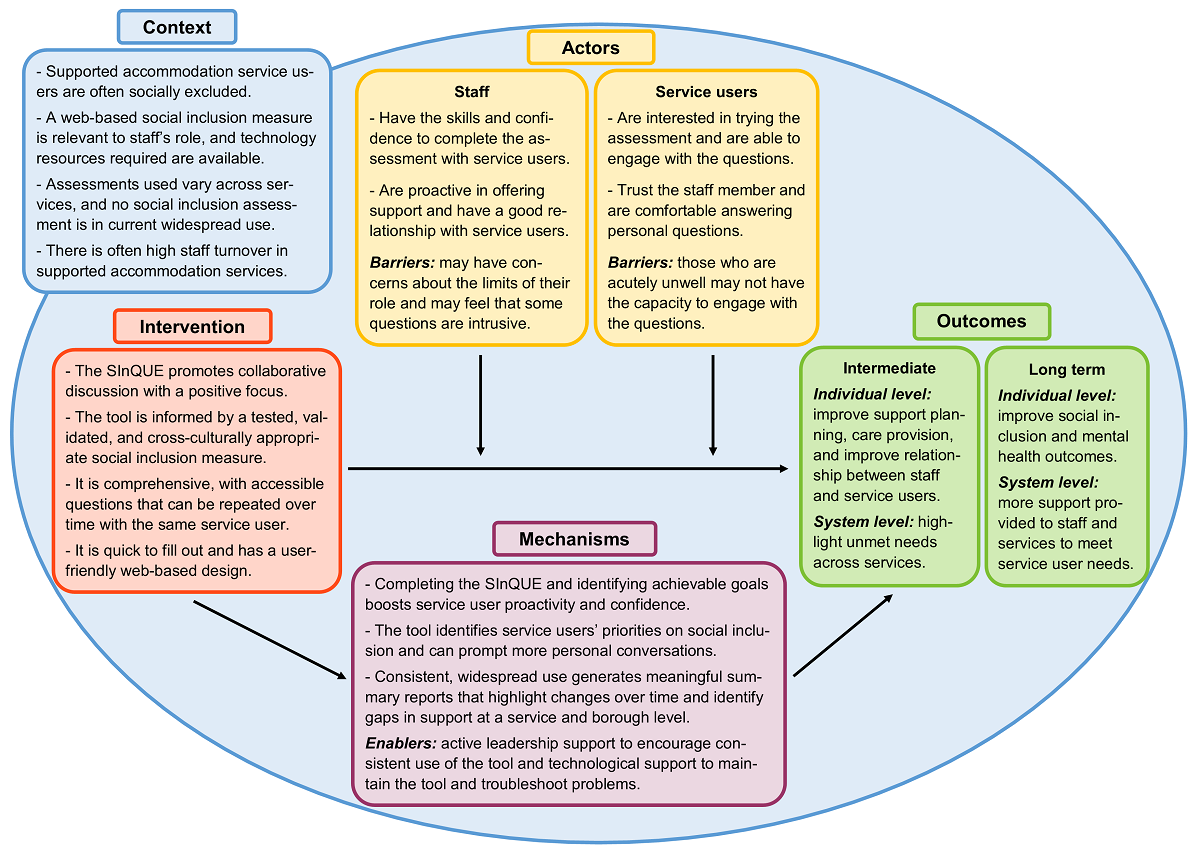
Intervention-context-actor-mechanism-outcome logic model for the online Social Inclusion Questionnaire User Experience (SInQUE).

On the basis of the collective study findings, we developed a logic model to summarize the processes involved in using the SInQUE and address study aim 4 (Figure 1).

The logic model was informed by the structure of an “ICAMO map” [38]. The model outlines the key aspects of the intervention (I), including its user-friendliness, its comprehensive nature, and the fact that it was based on a validated measure of social inclusion. It also indicates the potential outcomes (O) from using the SInQUE at both the individual and system levels, including improved support planning, better relationships, and provision of additional support for staff and services, which in turn may improve social inclusion and mental health outcomes as well as care provision more broadly. These operate within the broader societal context (C) of service users often being socially excluded and there being a high turnover of staff within these services and a high degree of variation across services in assessment tools that are recommended and in use, rendering the tool useful and relevant to the staff’s role.

The key actors (A) in implementing the SInQUE are the staff and service users, who require the skills and proactivity to administer the assessment and the motivation and trust to engage with the questions, respectively. Staff may encounter barriers such as a concern that some questions are too intrusive, and service users struggling more severely with their mental health may lack the concentration or motivation to engage with the questions. The potential outcomes operate through certain mechanisms (M), which include increased service user confidence and the prompting of more in-depth personal conversations between service users and staff. The tool also identifies more relevant priorities for service users, which may or may not be chosen as an active priority for support by staff owing to individual or organizational factors. Persistent and wide-ranging use of the tool could, over time, highlight the aspects of social inclusion that are feasible to work on and those that are regularly not being prioritized.

## Discussion

### Principal Findings

The online SInQUE was generally perceived as acceptable and potentially useful by supported accommodation staff and service users. This is consistent with findings of previous studies that used the SInQUE with other mental health populations [32,34]. Both staff and service users generally found the tool to be user-friendly and relevant and suggested that it could promote more targeted care planning and improve the relationship between staff and service users. Owing to the lower uptake of the SInQUE in residential care and floating outreach services, findings related to the tool’s utility in these settings are less conclusive than those for supported housing, where uptake was highest.

Some staff members expressed a concern that certain questions in the SInQUE could be perceived as intrusive by service users, indicating that they did not feel wholly comfortable asking what they perceived to be highly personal questions. However, this sentiment was not echoed by service users, who generally felt that the questions were appropriate and felt comfortable answering them. This finding is interesting given that supported accommodation service users have highlighted in previous research the importance of feeling personally understood by staff in their service and have endorsed a process of familiarization with staff [25].

We found that implementation support is essential to promote the use of the tool in services, as evidenced by the lack of use of the tool in the 2 regions where the SInQUE was introduced without a concerted implementation strategy. The most effective steps in our implementation strategy were those during which the use of the tool was actively endorsed by individuals in leadership positions, particularly service managers and local service leaders. However, even with our concerted implementation strategy, uptake of the SInQUE was only achieved with approximately 10% of service users living in mental health supported accommodation in the participating borough within the 5-month study period.

### Limitations

We used an established, iterative process of testing and feedback to develop the online SInQUE and determine its real-world acceptability and utility for use in mental health supported accommodation. However, it is important to acknowledge certain limitations of this study.

As mentioned previously, uptake of the SInQUE tool was highest in supported housing compared with residential care homes and floating outreach support. It is unclear whether this discrepancy reflects a greater reluctance from staff or service users in residential care and floating outreach support to use the online SInQUE. As proposed by one residential care staff member, it is possible that the staff in these services perceived the tool as being less relevant to their role. The discrepancy in part reflects the greater number of 24-hour and 9-to-5 supported housing units in the borough compared with residential care and floating outreach services, with approximately 6 times as many service users living in supported housing compared with residential care and nearly twice as many living in supported housing compared with floating outreach support. Through the local health service community mental health rehabilitation team, we also had a more direct connection with supported housing teams compared with other service types, which may have further contributed to the imbalance in services in which the SInQUE was used.

There were no female service user participants in the qualitative analysis; therefore, the findings may not be applicable to women in supported accommodation. It is unclear why it proved more difficult to recruit female participants, although it may reflect the higher proportion of male service users availing of supported accommodation in England—one review suggests that between 68% and 74% of service users are male across all supported accommodation types [40]. Furthermore, as we only tested the SInQUE in 1 London borough, the findings may not be generalizable to other regions.

As the tool was only used with approximately 10% of service users in the borough, the success of our implementation strategy was limited, and the low uptake may limit the wider generalizability of our findings. Owing to the short period and limited scope of the study, it was also not possible to assess whether use of the SInQUE in practice led to improved outcomes for service users or how useful the repeat assessments were over time. As the staff who participated volunteered to do so and they chose which service users to complete the SInQUE with, the findings may have been affected by selection bias and may not accurately reflect all supported accommodation staff members’ and service users’ views.

Finally, we removed 1 question from the original SInQUE questionnaire for our online version as asking people whether they lived alone was considered redundant for people living in supported housing. We also made very minor changes to the wording of 2 other questions in response to users’ feedback (Table 2). We think it is unlikely that these modest changes substantially affected the SInQUE’s psychometric properties. However, revalidation of the SInQUE in its web-based form is desirable in the future to confirm its validity and determine whether the minor wording changes should be retained for all versions of the SInQUE.

### Implications for Practice

The SInQUE can be recommended as a potentially useful and acceptable tool for use in mental health supported accommodation settings, particularly supported housing services that offer 24-hour or 9-to-5 support, to provide a thorough assessment of social inclusion and support care planning. The tool may help meet an identified wish from service users for more discussion and support with social inclusion and relationships [41]. It was evident during the study that there is currently no universal tool in widespread use to help with social inclusion in mental health supported accommodation, highlighting the potential gap for an assessment tool such as the SInQUE. If used widely across supported accommodation services, the online SInQUE has the potential to provide benchmarking data and identify service users’ most common priorities for greater social inclusion to inform service planning and evaluation.

Our findings also suggest that, for an assessment tool such as the SInQUE to be widely used, it is essential to have active leadership endorsement and support. For example, it may be required for managers or commissioners to direct staff to use the SInQUE with service users who are willing and reinforce this through team meetings, setting of use targets, or implementation of key performance indicators for its use.

### Implications for Research

It is important to hear from staff and service users who chose not to use the online SInQUE to understand their reasons for not using the tool and highlight barriers to using the tool that we may have missed in this study. It would be useful to conduct further testing of the tool in residential care and floating outreach supported accommodation settings to better determine the utility of the SInQUE in these service types. It would also be useful to examine the utility of the SInQUE in other population groups within different service types to determine whether the tool may be useful in additional settings.

Future research is necessary to establish the level of uptake of the SInQUE that can be achieved in supported accommodation over a longer period and potentially establish more effective means of implementation support. A longer-term study is also needed to establish whether the possible benefits from using the SInQUE that were mentioned by staff and service users are achievable through the use of the tool and how any potential outcomes may vary over time. A hybrid implementation-evaluation study would address these queries to determine the effectiveness of the SInQUE tool as an intervention for social inclusion and establish a precise implementation strategy for widespread uptake of the tool in supported accommodation. Further research using the SInQUE is also warranted to examine service user needs related to social inclusion and identify any additional barriers to addressing these needs in supported accommodation services. Such research could be used to inform the development of a future complex intervention to support social inclusion in supported accommodation services.

Although this study chose to examine the utility of the online SInQUE specifically in supported accommodation, the tool may also be useful in other mental health populations. Previous studies have established that the SInQUE can be used with mental health service users with a wide range of diagnoses [13,32,34]. Therefore, it is reasonable to extrapolate that the online SInQUE may be useful to assess social inclusion and inform support and care planning for other mental health service users, not just those living in mental health supported accommodation.

## Appendix

Multimedia Appendix 1 Full description of the online Social Inclusion Questionnaire User Experience using the TIDieR (Template for Intervention Description and Replication) checklist for reporting interventions.

Multimedia Appendix 2 Laboratory testing topic guide.

Multimedia Appendix 3 Field-testing topic guides (for staff and service users).

Multimedia Appendix 4 Explanation of suggested changes during laboratory testing and field-testing that were not made after team review.

Multimedia Appendix 5 Interviews with staff and service users—illustrative quotes for each inductively derived subtheme.

## Abbreviations

ICAMO: intervention-context-actor-mechanism-outcome
NHS: National Health Service
REC: Research Ethics Committee
SInQUE: Social Inclusion Questionnaire User Experience
TIDieR: Template for Intervention Description and Replication
UCL: University College London

## Data Availability

The qualitative data generated and analyzed during this study are not publicly available to preserve participant anonymity. The quantitative data generated during this study are available from the corresponding author (BLE) upon reasonable request.
